# Effects of Pyruvate Kinase M2 (PKM2) Gene Deletion on Astrocyte-Specific Glycolysis and Global Cerebral Ischemia-Induced Neuronal Death

**DOI:** 10.3390/antiox12020491

**Published:** 2023-02-15

**Authors:** Beom-Seok Kang, Bo-Young Choi, A-Ra Kho, Song-Hee Lee, Dae-Ki Hong, Min-Kyu Park, Si-Hyun Lee, Chang-Juhn Lee, Hyeun-Wook Yang, Seo-Young Woo, Se-Wan Park, Dong-Yeon Kim, Jae-Bong Park, Won-Suk Chung, Sang-Won Suh

**Affiliations:** 1 Department of Physiology, College of Medicine, Hallym University, Chuncheon 24252, Republic of Korea; 2 Department of Physical Education, Hallym University, Chuncheon 24252, Republic of Korea; 3 Institute of Sport Science, Hallym University, Chuncheon 24252, Republic of Korea; 4 Neuroregeneration and Stem Cell Programs, Institute for Cell Engineering, College of Medicine, Johns Hopkins University School, Baltimore, MD 21205, USA; 5 Department of Neurology, College of Medicine, Johns Hopkins University School, Baltimore, MD 21205, USA; 6 Department of Pathology and Laboratory Medicine, College of Medicine, Emory University School, Atlanta, GA 30322, USA; 7 Department of Biochemistry, College of Medicine, Chuncheon 24252, Republic of Korea; 8 Department of Biological Sciences and KAIST Stem Cell Center, Korea Advanced Institute of Science and Technology, Daejeon 34051, Republic of Korea

**Keywords:** global cerebral ischemia, astrocyte–neuron lactate shuttle, pyruvate kinase M2, sodium l-lactate, neuronal death

## Abstract

Ischemic stroke is caused by insufficient blood flow to the brain. Astrocytes have a role in bidirectionally converting pyruvate, generated via glycolysis, into lactate and then supplying it to neurons through astrocyte–neuron lactate shuttle (ANLS). Pyruvate kinase M2 (PKM2) is an enzyme that dephosphorylates phosphoenolpyruvate to pyruvate during glycolysis in astrocytes. We hypothesized that a reduction in lactate supply in astrocyte PKM2 gene deletion exacerbates neuronal death. Mice harboring a PKM2 gene deletion were established by administering tamoxifen to Aldh1l1-Cre^ERT2^; PKM2^f/f^ mice. Upon development of global cerebral ischemia, mice were immediately injected with sodium l-lactate (250 mg/kg, i.p.). To verify our hypothesis, we compared oxidative damage, microtubule disruption, ANLS disruption, and neuronal death between the gene deletion and control subjects. We observed that PKM2 gene deletion increases the degree of neuronal damage and impairment of lactate metabolism in the hippocampal region after GCI. The lactate administration groups showed significantly reduced neuronal death and increases in neuron survival and cognitive function. We found that lactate supply via the ANLS in astrocytes plays a crucial role in maintaining energy metabolism in neurons. Lactate administration may have potential as a therapeutic tool to prevent neuronal damage following ischemic stroke.

## 1. Introduction

Ischemic stroke is a major brain disease that affects people worldwide; it is associated with complex problems and is caused by hemorrhage or cerebral infarction [1]. Consequently, ischemic stroke causes severe neuronal damage in the brain along with cognitive impairment. Progression of neuronal death is caused by insufficient supply of oxygen, glucose, and nutrients to the brain resulting from cerebrovascular infarction [2]. Cerebral ischemia can be divided into two main types: global and focal. Sudden cardiac arrest (CA) induces drastically decreased blood flow, and most cases of global cerebral ischemic followed by CA and brain damage affect wide regions of the brain. Focal cerebral ischemia is caused by blocking blood flow, particularly in the brain [3,4,5]. In addition, the restoration of reduced and blocked blood flow to the brain leads to secondary brain damage, which is called “reperfusion injury”. To restore ischemic brain damage, thrombolysis or resuscitation is necessary, although they can cause reperfusion injury. During reperfusion, reactive oxygen species (ROS) are produced and neuroinflammation is activated. Subsequently, the blood–brain barrier (BBB) is disrupted followed by neuronal cell death cascades [6].

Pyruvate kinase (PK) is an enzyme that is required for the final step of glycolysis, wherein it dephosphorylates phosphoenolpyruvate (PEP) to pyruvate and phosphorylates ADP to ATP [7]. PK has four isoforms: PKM1, PKM2, PKR, and PKL [8]. PKM1 and PKM2 are selectively produced by alternative splicing of the PKM pre-mRNA, which involves either exon 9 (PKM1) or exon 10 (PKM2) [9]. In the brain, it has been shown that PKM1 is highly expressed in neurons, while PKM2 is specific to astrocytes [10]. PKM2 not only mediates glucose metabolism to generate pyruvate and ATP in the cytoplasm but also causes a Warburg effect in cancer cells [11]. Moreover, it is known that PKM2 is required for cell proliferation and cell death through interacting with various transcription factors including β-catenin, octamer-binding transcription factor 4 (Oct-4), signal transducer and activator of transcription 3 (STAT3), and hypoxia-induced factor 1 (HIF-1) [9]. In particular, PKM2 interacting directly with HIF-1α accelerates transactivation of HIF-1α target genes, including lactate dehydrogenase A (LDHA) [12]. Thus, it was inferred that PKM2 mainly contributes to lactate production in astrocytes [13].

Glucose is the main energy source in the central nervous system (CNS) for producing ATP in neurons [14]. Glucose is transferred from brain vessels to astrocytes via a glucose transporter and then converted into pyruvate and lactate through PKM2 and LDHA. Converted lactate in astrocyte moves to nearby neurons via monocarboxylate transporters (MCTs) to supply ATP substrates. MCT1, -2, and -4 are mainly expressed in the brain; in particular, MCT1 localizes to endothelial cells of the BBB in rodents and humans [15,16]. Supplying lactate for energy production from astrocytes to neurons is mainly conducted through MCT2 and MCT4. The high-capacity and low-affinity transporter MCT4 in astrocytes delivers lactate from astrocytes to neurons. In contrast, MCT2 is known to be generally expressed in neurons and transfers lactate secreted from astrocytes to the neuronal cytoplasm [17,18]. MCT3 is constricted to cells in the choroid plexus epithelium and retinal pigment [19]. In neurons, the lactate supplied from astrocytes is converted back into pyruvate by LDHB, which is transferred into mitochondria for aerobic energy production through the tricarboxylic acid (TCA) cycle [20]. Thus, glucose absorbed by astrocytes is converted to lactate and then delivered to neurons for use as a neuronal energy source, a process that is referred as the astrocyte–neuron lactate shuttle (ANLS) hypothesis [21].

Under pathophysiological conditions such as hypoglycemia, lactate is known as a potential substrate that replaces glucose in the brain [22,23]. Several previous studies have demonstrated that lactate can protect neurons against brain damage. Uehara and Suzuki et al. reported that lactate is converted from glucose by the stimulation of various neurotransmitter receptors and contributes to memory formation and cognitive functions [24,25]. Oddo et al. verified that hyperglycolysis with lactate production preserves subarachnoid hemorrhage [26]. Holloway, Ros, and Annoni et al. showed that, in cases of traumatic brain injury, the intracerebroventricular administration of lactate has a neuroprotective effect against glutamate-induced excitotoxicity [27,28,29]. Through the above studies, it appears that lactate can be used as a substitute for glucose during abnormal energy metabolism under various pathological conditions.

Given that lactate supplementation is useful in energy metabolism, we hypothesized that deletion of the PKM2 gene in astrocytes may exacerbate neuronal death by reducing the supply of lactate from astrocytes to neurons via ANLS in the case of GCI. Moreover, lactate supplementation after GCI may have a neuroprotective effect even after blockage of ANLS. To demonstrate our hypothesis, we genetically deleted the astrocyte-specific PKM2 gene using Aldh1l1-Cre^ERT2^; PKM2 floxed mice. Then, we investigated the differences in neuronal death, reactive oxygen species production, and microtubule damage following GCI in PKM2 wild-type and -deleted mice. Furthermore, we investigated whether there are differences in lactate metabolism-related factors such as LDHA, LDHB, and MCT4 after GCI with PKM2 gene deletion. Finally, we administrated additional lactate to demonstrate the neuroprotective effects of lactate supplementation. Here, we found an increase in neuronal death after GCI in PKM2 gene-deleted mice, a phenomenon that could be rescued by lactate supplement. Consequently, the results of the present study suggest that lactate supplementation for enhanced energy metabolism activity via ANLS plays a main role in neuronal survival and is a potential new therapeutic tool for preventing neuronal death after ischemia.

## 2. Materials and Methods

### 2.1. Animal Care Management and Ethics Statement

The experimental animals received care according to the protocol of the Hallym University Animal Research Committee (protocol #Hallym 2020-38, 20 October 2020). PKM2^f/f^ and Aldh1l1-Cre^ERT2^; PKM2^f/f^ mice (background strains, C57BL/6J), adult males, aged 2–3 months, were bred and conserved in the facility of Hallym University College of Medicine. The animals were kept under continuous environmental conditions at 3 to 4 mice per cage (humidity 55% ± 5%, temperature 20 ± 2 °C, 12 h/12 h light–dark) and given standard feed and water by Purina (Gyeonggi-do, Korea) ad libitum. We administered isoflurane anesthesia at 2–3% to minimize pain during animal experiments. In addition, this manuscript was prepared according to the standards set out in ARRIVE (Animal in Research: Reporting In Vivo Experiments) [30].

### 2.2. PCR Genotyping

Toe clippings from 4-week-old mice were used to extract DNA for PCR genotyping with 2 primers. DNA was extracted from the mouse toe clippings using DNA kits (Bioneer lnc., Daejeon, Korea). PCR genotyping was confirmed with primers set before all experiments (Aldh1l1-Cre^ERT2^ forward (5′-CTTCAACAGGTGCCTTCCA-3′), Aldh1l1-Cre^ERT2^ reverse (5′-GGCAAACGGACAGAAGCA-3′)). The Aldh1l1-Cre^ERT2^ band is 198 bp in size. PCR was performed with 2% agarose gel electrophoresis and imaged through use of ethidium bromide.

### 2.3. Tamoxifen and Lactate Administration

Tamoxifen (Sigma-Aldrich Co., St. Louis, MO, USA) was melted in corn oil (Sigma-Aldrich Co., St. Louis, MO, USA) to 20 mg/mL. Mice (4 weeks old) were intraperitoneally administered with 75 mg tamoxifen/kg weight for 5 consecutive days. Tamoxifen was administered to both Aldh1l1-Cre^ERT2^; PKM2^f/f^ and PKM2^f/f^ groups. The GCI induction was performed 4–5 weeks after tamoxifen administration.

To confirm the effects of the drug, post-administration, in reducing GCI-induced neuronal death, we used sodium l-lactate (Sigma-Aldrich Co., St. Louis, MO, USA), which was dissolved in 0.9% saline for use in the experiment and intraperitoneally administered at 250 mg/kg weight immediately after GCI. Mice of all experimental groups were sacrificed 3 days after GCI.

### 2.4. Mouse Global Cerebral Ischemia

Male mice (weight 25–30 g, aged 2–3 months) were GCI-induced 4–5 weeks after tamoxifen injection. Mice were anesthetized by inhalation using 2–3% isoflurane in a 25:75 mix of oxygen and nitrogen. The operation was performed by checking the homoeothermic monitoring system to maintain the core body temperature of the mouse at 36.5–37.5 °C (Harvard Apparatus, Holliston, MA, USA). The neck midline of the anesthetized mice was incised, and the bilateral common carotid arteries (BCCAs) were isolated. BCCAs were occluded for 20 min using aneurysmal clips. Isoflurane was lowered to 1% during the occlusion period [31]. After 20 min of ischemia, the aneurysmal clips were removed, and normal recovery of the mouse was confirmed. The spontaneous respiration of mice was confirmed, which recovered in an animal incubator at 37 °C.

### 2.5. Preparing Brain Tissue Samples

GCI-induced mice were anesthetized using urethane (1.5 g/kg, i.p.) and sacrificed on day 3. The sacrificed mice were perfused with saline to drain whole blood. Brain harvesting in mice was performed after being fixed using 4% paraformaldehyde (PFA). After brain harvesting, it was post-fixed for approximately in 4% PFA. When post-fixing was finished, PFA was replaced with 30% sucrose solution and left until the brain sank. The brain was then frozen, and sections of 30 μm thickness were prepared using a cryostat microtome (CM1850, Leica, Wetzlar, Germany).

### 2.6. Confirmation of Neuronal Death

In the present study, Fluoro-Jade B (FJB) staining was conducted 3 days after GCI induction to confirm neuronal death. The brain tissues were placed on gelatin-coated slides (Fisher Scientific, Pittsburgh, PA, USA) and stained with FJB according to the method used in Kang [31]. The stained slides were covered with a cover slide using DPX (Sigma-Aldrich Co., St. Louis, MO, USA), and staining confirmed using a fluorescence microscope (SZ61, Olympus, Shinjuku, Japan, FITC green fluorescence excitation—light wavelength: 450–490 nm). Around five to six coronal brain sections were collected for blind counting of the FJB-positive cells. The FJB + cells were investigated in the hippocampal regions. The significance in the FJB data was confirmed using statistical analysis.

### 2.7. Immunofluorescence Analysis

For immunostaining, the brain tissue was pretreated with incubation in 1.2% hydrogen peroxide for 15 min followed by washing in 0.01% PBS. Tissues were stained with several primary antibodies diluted in PBS containing 0.3% Triton X-100. The primary antibodies used in this study are as follows: PKM2 (diluted 1:500, Cell Signaling Technologies, Danvers, MA, USA); S100B (diluted 1:500, Synaptic Systems, Goettingen, Germany); 4-HNE (diluted 1:500, Alpha Diagnostic Intl. Inc., San Antonio, TX, USA); MAP2 (diluted 1:200, Alpha Diagnostic Intl. Inc.); LDHA (diluted 1:100, Sigma-Aldrich Co., St. Louis, MO, USA); LDHB (diluted 1:100, Sigma-Aldrich Co.); NeuN (diluted 1:500, Billerica, Millipore Co., Burlington, MA, USA); MCT4 (1:150, Santa Cruz Biotechnology, Dallas, CA, USA); MCT2 (1:1000, Invitrogen, Waltham, MA, USA). Then, we washed the brain tissue with 0.01 M PBS and stained with the appropriate secondary antibody (Alexa-Fluor-594-conjugated IgG secondary antibody and Alexa-Fluor-488-conjugated secondary antibody, both diluted 1:250, Invitrogen, Grand Island, NY, USA). To confirm staining, brain tissue was examined under a microscope using gelatin-coated slides. The ImageJ program (NIH, Bethesda, Rockville, MD, USA) was used to analysis the intensity [3,31].

### 2.8. Immunohistochemistry

Similar to immunofluorescence analysis above, the pretreatment process was conducted before incubation with the primary antibody NeuN (diluted 1:500, Billerica, Millipore Co., Burlington, MA, USA) for attachment. After pretreatment processing, the brain tissues were stained with anti-rabbit IgG antibody (diluted 1:250, Jackson Immunoresearch lac, West Grove, PA, USA) in PBS containing 0.3% Triton X-100 for 2 h at RT. Then, after washing with PBS, the brain tissues were placed in ABC solution (Vector Laboratories, Burlingame, Vector, CA, USA) for 2 h. After washing with PBS, the samples were incubated with a solution of DAB (0.06% 3, 3′-diaminobenzidine solution, Sigma-Aldrich Co., St Louis, MO, USA) in 0.01 M PBS containing H2O2 for 1 min 30 s. To confirm staining, brain tissue was examined under a microscope using gelatin-coated slides. The ImageJ program (NIH, Bethesda, Rockville, MD, USA) was utilized to measure the intensity [32].

### 2.9. Behavioral Test

#### 2.9.1. Neurological Severity Score (NSS) Test

To test whether lactate administration improved GCI-induced neurologic motor function in Aldh1l1-Cre^ERT2^; PKM2^f/f^, we calculated the 10-point neurological severity score (NSS) as explained by Tsenter et al. [33]. These tests were evaluated continuously 7 days after GCI or sham surgery. The NSS test comprises 10 different tasks for which general behavior, balance, alertness, and motor ability are estimated. If the mice fail a task, they receive 1 point. Therefore, a score closer to 0 points indicates a healthier mouse that is successful in all tasks, whereas a score closer to 10 points indicates a severely damaged mouse [34] (Table S1).

#### 2.9.2. Morris Water Maze Test

To establish whether lactate administration allowed recovery of GCI-induced cognitive dysfunction in Aldh1l1-Cre^ERT2^; PKM2^f/f^, we performed the MWM test from 8 to 13 days after GCI. The MWM test involves use of a circular water tank (1 m diameter) filled with water (30 cm diameter, 22–26 °C) that consists of four equal quadrants. A hidden platform is set in the center of one quadrant, submerged 1 cm below the water surface. Mice start in four different zones for each trial, and the test comes to an end when the mice find the hidden platform. In each trial, the test mice swam in the water for a maximum of 120 s to find the hidden platform. We measured this using a camera and the SMART video tracking software 3.0 (Panlab, Carrer de l’Energia, Spain), capturing the time between when the mice found the hidden platform and when they dropped into the water in a zone [32,35].

### 2.10. Statistical Analysis

All statistical data from this study were measured using ImageJ (National Institutes of Health, Bethesda, MD, USA) and presented as the mean value ± SEM. Nonparametric testing between groups was performed using the Mann–Whitney U test or Kruskal–Wallis test with post hoc Bonferroni correction. Behavioral data were assessed for variance (ANOVA). A value of *p* < 0.05 indicates a statistically significant difference. All data were analyzed using IBM the Statistical Package for the Social Sciences (IBM SPSS statisics version 25, Chicago, IL, USA) software.

## 3. Results

### 3.1. PKM2 Gene Deletion Reduces the Expression of PKM2 in Astrocytes

**Figure 1:**
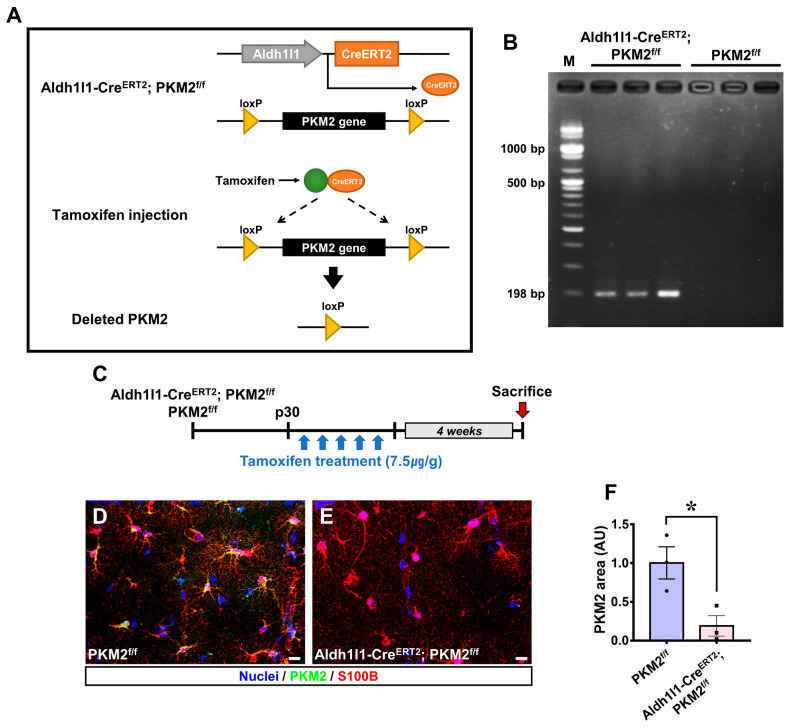
(**A**) Tamoxifen-induced PKM2 gene deletion was confirmed by PCR genotyping and immunofluorescence in the cornus ammonis 1 (CA1) region. Schematic structure in which the PKM2 gene is deleted from Aldh1l1-Cre^ERT2^; PKM2^f/f^ after administration of tamoxifen. (**B**) PCR genotyping of representative showed mice with a 198 bp PCR product specifically for the Aldh1l1-Cre^ERT2^; PKM2^f/f^ gene (Aldh1l1-Cre^ERT2^; PKM2^f/f^, PKM2 (-/-); PKM2^f/f^, PKM2 wild type). (**C**) Schematic representation showing that Aldh1l1-Cre^ERT2^; PKM2^f/f^ group and PKM2^f/f^ group were sacrificed 4 weeks after administration of tamoxifen (7.5 μg/g, i.p.) for 5 days once daily after p30. (**D**,**E**) Representative image showing expression of PKM2 (green) and S100B (astrocyte marker, red) in the CA1 regions of the hippocampus. Scale bar = 50 μm. (**F**) Graph showing the PKM2 area (AU) intensity in the CA1 region. The PKM2 area was decreased in the Aldh1l1-Cre^ERT2^; PKM2^f/f^ group (●PKM2 ^f/f^, *n* = 3; ■Aldh1l1-Cre^ERT2^; PKM2^f/f^, *n* = 3). * Significantly different from the PKM2^f/f^ group, Aldh1l1-Cre^ERT2^; PKM2^f/f^ group, *p* < 0.05. (Bonferroni post hoc test after Kruskal–Wallis test: *p* = 0.050, df = 1, chi square = 3.857). Data are shown as mean ± SEM.

To investigate whether Aldh1l1-Cre^ERT2^; PKM2^f/f^ mice had reduced expression of PKM2 in astrocytes (Figure 1A), tamoxifen (75 mg/kg) was administered to Aldh1l1-Cre^ERT2^; PKM2^f/f^ and PKM2^f/f^ mice once a day for 5 days after a postnatal period of 30 days (Figure 1C). The differences between Aldh1l1-Cre^ERT2^; PKM2^f/f^ mice and PKM2^f/f^ mice are determined by the presence or absence of the Aldh1l1-Cre^ERT2^ gene, which was confirmed by PCR genotyping (Figure 1B). The mice were sacrificed 4 weeks after tamoxifen administration. To assess the knockout of PKM2 in astrocytes, we performed immunofluorescence staining of PKM2 (green) and S100B (astrocyte marker, red) in the hippocampal cornus ammonis 1 (CA1) (Figure 1D,E). PKM2-positive intensity in astrocytes was decreased in the Aldh1l1-Cre^ERT2^; PKM2^f/f^ group compared with the PKM2^f/f^ group by about 81% in the CA1 region (PKM2^f/f^, 1.00 ± 0.21; Aldh1l1-Cre^ERT2^; PKM2^f/f^, 0.19 ± 0.13) (Figure 1F).

### 3.2. PKM2 Gene Deletion Exacerbates Hippocampal Neuron Death after GCI

**Figure 2:**
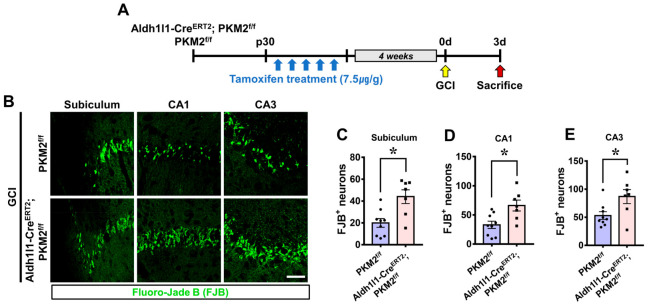
(**A**) Schematic representation of the experimental timeline showing that mice of the GCI-Aldh1l1-Cre^ERT2^; PKM2 f/f group, and the GCI-PKM2^f/f^ group were sacrificed 3 days after GCI insult. (**B**) Fluorescent images indicate degenerated neurons in the subiculum, CA1, and CA3 regions. More degenerating neurons (FJB, green) 3 days after ischemia can be seen in the GCI-Aldh1l1-Cre^ERT2^; PKM2^f/f^ group compared with the GCI-PKM2^f/f^ group. Scale bar = 100 μm. (**C**–**E**) The graph displays quantification of the GCI-Aldh1l1-Cre^ERT2^; PKM2^f/f^ group compared with the GCI-PKM2^f/f^ group (●PKM2^f/f^ group, *n* = 9; ■Aldh1l1-Cre^ERT2^; PKM2^f/f^ group, *n* = 7). * Significantly different from the PKM2^f/f^ group, Aldh1l1-Cre^ERT2^; PKM2^f/f^ group, *p* < 0.05. Data are shown as mean ± SEM (Mann–Whitney U test measurement results, subiculum: z = 2.488, *p* = 0.013; CA1: z = 2.382, *p* = 0.017; CA3: z = 2.711, *p* = 0.007).

Neuronal death caused by global cerebral ischemia (GCI) was confirmed and measured through Fluoro-Jade B (FJB) staining. The experimental timeline shows that GCI was induced 4 weeks after tamoxifen administration, and the mice were sacrificed at 3 days following GCI (Figure 2A). Degenerating neurons found in the subiculum, CA1, and CA3 regions were compared between the GCI-Aldh1l1-Cre^ERT2^; PKM2^f/f^ group and the GCI-PKM2^f/f^ group (Figure 2B). The GCI-Aldh1l1-Cre^ERT2^; PKM2^f/f^ group displayed an increase in FJB+ neurons of about 54% in the subiculum (GCI-Aldh1l1-Cre^ERT2^; PKM2^f/f^, 43.9 ± 6.2; GCI-PKM2^f/f^, 19.9 ± 3.9), 50% in the CA1 (GCI-Aldh1l1-Cre^ERT2^; PKM2^f/f^, 66.0 ± 9.3; GCI-PKM2^f/f^, 32.9 ± 6.2) and 39% in the CA3 (GCI-Aldh1l1-Cre^ERT2^; PKM2^f/f^, 86.7 ± 12.4; GCI-PKM2^f/f^, 52.9 ± 6.8) regions compared with the GCI-PKM2^f/f^ group (Figure 2C–E).

### 3.3. PKM2 Gene Deletion Increases Disturbance of Microtubule Structure after GCI

**Figure 3:**
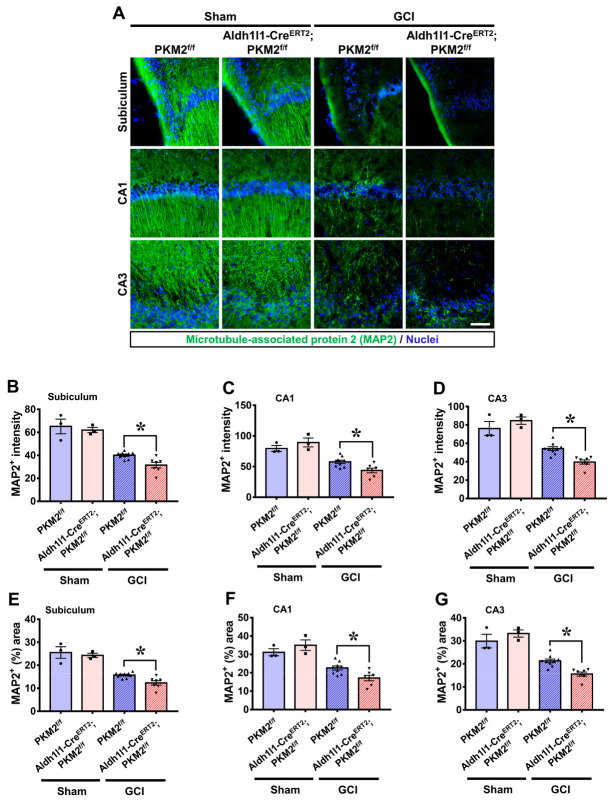
(**A**) Microtubules visualized through staining of microtubule-associated protein 2 (MAP2, green) in the subiculum, CA1, and CA3 regions 3 days after GCI. Scale bar = 100 μm. (**B**–**G**) Graph showing the MAP2+ intensity and percent area in the subiculum, CA1, and CA3. The GCI groups shows that there is lower MAP2+ staining intensity and (%) area in the Aldh1l1-Cre^ERT2^; PKM2^f/f^ group compared with the PKM2^f/f^ group (●Sham-PKM2^f/f^ group, *n* = 3; ■Sham-Aldh1l1-Cre^ERT2^; PKM2^f/f^, *n* = 3; ▲GCI-PKM2^f/f^ group, *n* = 9, ▼GCI-Aldh1l1-Cre^ERT2^; PKM2^f/f^, *n* = 7). * Significantly different from the PKM2^f/f^ group, Aldh1l1-Cre^ERT2^; PKM2^f/f^ group, *p* < 0.05. Data are shown as mean ± SEM (Bonferroni post hoc test after Kruskal–Wallis test, subiculum: chi square = 17.112, df = 3, *p* = 0.001; CA1: chi square = 16.236, df = 3, *p* = 0.001; CA3: chi square = 18.160, df = 3, *p* < 0.001).

To investigate whether GCI-induced brain structural damage is associated with degeneration in neuronal processes and microtubule dissociation in both PKM2^f/f^ and PKM2 deleted mice, we stained microtubule-associated protein 2 (MAP2) antibodies to assess possible microtubule disruption. Intact MAP2-positive microtubule structures were observed in the hippocampal regions of Sham group mice. However, the GCI-subjected PKM2 deletion mice had lower MAP2-positive intensity, which covered a smaller area compared with in PKM2^f/f^ mice (Figure 3A). Microtubule staining intensity and distribution area were lower in the GCI-Aldh1l1-Cre^ERT2^; PKM2^f/f^ group compared with the GCI-PKM2^f/f^ group by about 21% in the hippocampal subiculum (GCI-PKM2^f/f^, 40.1 ± 0.9; GCI-Aldh1l1-Cre^ERT2^; PKM2^f/f^, 31.4 ± 2.4), 24% in the CA1 region (GCI-PKM2^f/f^, 57.6 ± 2.7; GCI-Aldh1l1-Cre^ERT2^; PKM2^f/f^, 43.6 ± 3.7), and 27% in the CA3 region (GCI-PKM2^f/f^, 54.3 ± 2.0; GCI-Aldh1l1-Cre^ERT2^; PKM2^f/f^, 39.6 ± 2.2) (Figure 3B–G).

### 3.4. PKM2 Gene Deletion Increases Oxidative Damage after GCI

**Figure 4:**
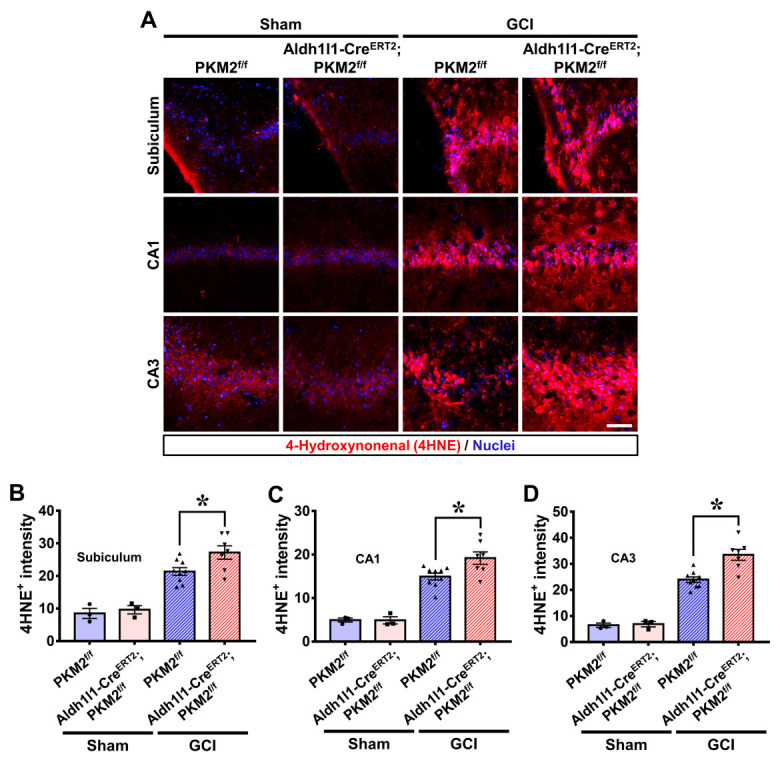
(**A**) Lipid peroxidation shown by staining of 4-hydroxynonenal (4-HNE, red) in the subiculum, CA1, and CA3 regions 3 days after GCI. Scale bar = 100 μm. (**B**–**D**) Graph shows the average fluorescence intensities of 4-HNE + in the subiculum, CA1, and CA3 regions. For the GCI groups, Aldh1l1-Cre^ERT2^; PKM2^f/f^ group has higher 4-HNE+ staining intensity compared with the PKM2^f/f^ group (●Sham-PKM2^f/f^ group, *n* = 3; ■Sham-Aldh1l1-Cre^ERT2^; PKM2^f/f^, *n* = 3; ▲GCI-PKM2^f/f^ group, *n* = 9, ▼GCI-Aldh1l1-Cre^ERT2^; PKM2^f/f^, *n* = 7). * Significantly different from the PKM2^f/f^ group, Aldh1l1-Cre^ERT2^; PKM2^f/f^ group, *p* < 0.05. Data are shown as mean ± SEM (Bonferroni post hoc test after Kruskal–Wallis test, subiculum: chi square = 15.341, df = 3, *p* = 0.002; CA1: chi square = 15.310, df = 3, *p* = 0.002; CA3: chi square = 16.755, df = 3, *p* = 0.001).

To confirm whether GCI-induced reactive oxygen species (ROS) cause damage, we used 4-hydroxynonenal (4-HNE) staining to detect lipid peroxidation. The mice were sacrificed at 3 days after GCI. Histological evaluation of lipid peroxidation with 4-HNE was measured in the hippocampal regions. The sham group indicated no difference in the 4-HNE signal. However, the GCI groups showed a drastic increase in 4-HNE fluorescence signal. In particular, the GCI-Aldh1l1-Cre^ERT2^; PKM2^f/f^ group showed a significantly increased 4-HNE intensity compared with the GCI-PKM2^f/f^ group (Figure 4A). Lipid peroxidation was higher in the GCI-Aldh1l1-Cre^ERT2^; PKM2^f/f^ group compared with the GCI-PKM2^f/f^ group by about 21% in the subiculum (GCI-PKM2^f/f^, 21.3 ± 1.1; GCI-Aldh1l1-Cre^ERT2^; PKM2^f/f^, 27.1 ± 2.0), 22% in the CA1 (GCI-PKM2^f/f^, 14.9 ± 0.7; GCI-Aldh1l1-Cre^ERT2^; PKM2^f/f^, 19.1 ± 1.4), and 28% in the CA3 (GCI-PKM2^f/f^, 23.9 ± 1.0; GCI-Aldh1l1-Cre^ERT2^; PKM2^f/f^, 33.4 ± 2.0) regions (Figure 4B–D).

### 3.5. PKM2 Gene Deletion Reduces the Expression of MCT4, LDHA, and LDHB in the Hippocampus after GCI

**Figure 5:**
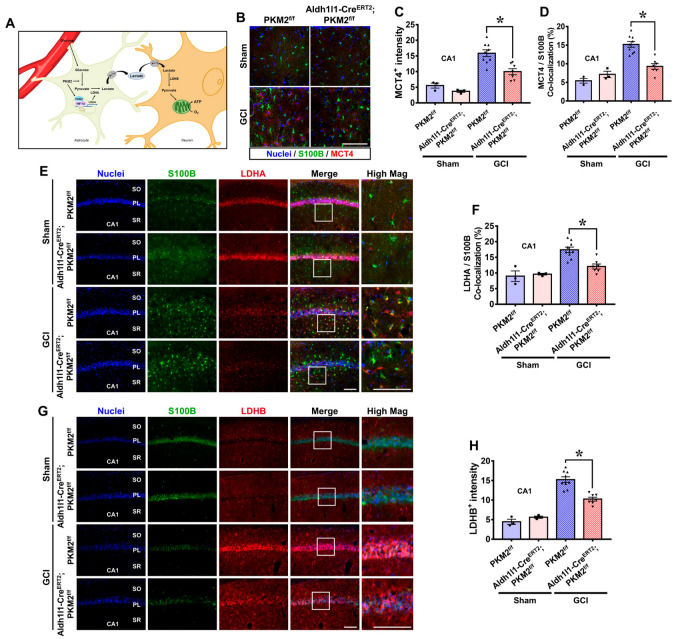
(**A**) Schematic illustration depicting the astrocyte–neuron lactate shuttle hypothesis for PKM2 wild type and PKM2 gene deletion after GCI. (**B**) PKM2 gene deletion reduces monocarboxylate transporter 4 (MCT4) in astrocytes after 3 days of GCI. Fluorescent image indicates MCT4 (red), S100B (astrocyte marker, green), and merged image in the hippocampal CA1 region at 3 days after GCI. Scale bar = 100 μm. (**C**,**D**) Graph showing the average MCT4+ intensity and MCT4 and S100B co-localization intensity in CA1. The GCI groups show reduced MCT4 and co-localization in the GCI-Aldh1l1-Cre^ERT2^; PKM2^f/f^ group compared with the GCI-PKM2^f/f^ group (Sham-PKM2^f/f^ group, *n* = 3; Sham-Aldh1l1-Cre^ERT2^; PKM2^f/f^, *n* = 3; GCI-PKM2^f/f^ group, *n* = 9, GCI-Aldh1l1-Cre^ERT2^; PKM2^f/f^, *n* = 7). (**E**) Fluorescent image showing LDHA (red), S100B (astrocyte marker, green), and merged image in the CA1 region at 3 days after GCI. Scale bar = 100 μm. (**F**) Bar graph showing LDHA and S100B co-localization (%) in the CA1. The GCI groups indicate reduced LDHA and S100B co-localization in the Aldh1l1-Cre^ERT2^; PKM2^f/f^ group compared with the PKM2^f/f^ group (Sham-PKM2^f/f^ group, *n* = 3; Sham-Aldh1l1-Cre^ERT2^; PKM2^f/f^, *n* = 3; GCI-PKM2^f/f^ group, *n* = 9, GCI-Aldh1l1-Cre^ERT2^; PKM2^f/f^, *n* = 7). (**G**) PKM2 gene deletion reduces lactate dehydrogenase B (LDHB) in living neurons after 3 days of GCI. Fluorescent image showing LDHB (red), NeuN (living neurons, green), and merged image in the CA1 regions at 3 days after GCI. Scale bar = 100 μm. (**H**) The graph shows the average of LDHB+ intensity in the CA1. The GCI groups indicates that the Aldh1l1-Cre^ERT2^; PKM2^f/f^ group reduced LDHB compared to the PKM2^f/f^ group (Sham-PKM2^f/f^ group, *n* = 3; Sham-Aldh1l1-Cre^ERT2^; PKM2^f/f^, *n* = 3; GCI-PKM2^f/f^ group, *n* = 9, GCI-Aldh1l1-Cre^ERT2^; PKM2^f/f^, *n* = 7). (●Sham-PKM2^f/f^ group; ■Sham-Aldh1l1-Cre^ERT2^; PKM2^f/f^; ▲GCI-PKM2^f/f^ group; ▼GCI-Aldh1l1-Cre^ERT2^; PKM2^f/f^). * Significantly different from the PKM2^f/f^ group, Aldh1l1-Cre^ERT2^; PKM2_f/f_ group, *p* < 0.05. Data are shown as mean ± SEM (Bonferroni post hoc test after Kruskal–Wallis test, MCT4: chi square = 17.513, df = 3, *p* = 0.001; MCT4/S100B: chi square = 16.819, df = 3, *p* = 0.001; LDHA/S100B: chi square = 16.100, df = 3, *p* = 0.001; LDHB: chi square = 18.597, df = 3, *p* < 0.001).

The astrocyte–neuron lactate shuttle (ANLS) hypothesis indicates that glucose absorbed by astrocytes is converted to pyruvate through the glycolysis process, and lactate converted through lactate dehydrogenase A (LDHA) is then transferred to neurons through monocarboxylate transporter 4 (MCT4). Lactate transferred to neurons through MCT2 is transformed back to pyruvate and used as an energy source to produce ATP via mitochondria (Figure 5A). We showed CA1 as representative images and data since it is the most vulnerable hippocampal regions in the brain after ischemia [36,37]. MCT4 is one of the transporters that releases lactate from astrocytes in the gaps between astrocytes and neuronal space. To examine the expression of MCT4, we conducted double staining of MCT4 (red) and S100B (astrocyte marker, green). No differences in MCT4 fluorescence signals were observed in the sham groups. Much higher MCT4 fluorescence signal was observed in the GCI groups compared with the sham groups. However, the GCI-Aldh1l1-Cre^ERT2^; PKM2^f/f^ group had significantly reduced MCT4 expression compared with the GCI-PKM2^f/f^ group (Figure 5B). MCT4 and S100B co-localization was lower in the GCI-Aldh1l1-Cre^ERT2^; PKM2^f/f^ group compared with the GCI-PKM2^f/f^ group, by about 37% and 39% in the CA1 region (GCI-PKM2^f/f^, 15.8 ± 1.0 and 15.2 ± 2.3; GCI-Aldh1l1-Cre^ERT2^; PKM2^f/f^, 9.9 ± 0.9 and 9.2 ± 1.9) (Figure 5C,D). MCT2 is a type of transporter that takes up extracellular lactate. To evaluate the expression of MCT2 in neurons, we identified live neurons and MCT2 staining in the hippocampal region (Figure S1A). MCT2 expression did not differ between the sham groups but was comparatively lower in the GCI groups. Moreover, in the GCI groups, MCT2 expression was significantly reduced by 23% in GCI-Aldh1l1-CreERT2; PKM2f/f compared with GCI-PKM2f/f (GCI-PKM2^f/f^, 11.8 ± 1.6; GCI-Aldh1l1-Cre^ERT2^; PKM2^f/f^, 9.1 ± 1.3) (Figure S1B). LDHA is an enzyme that converts pyruvate to lactate in astrocyte. To investigate GCI-induced LDHA overexpression in astrocytes, we performed LDHA (red) and S100B (astrocyte marker, green) double staining at 3 days after GCI. We found that co-localized LDHA expression was specifically localized in astrocytes. There were no differences in LDHA and S100B co-localized fluorescence signals in each sham group. The GCI groups had significantly increased LDHA and S100B co-localized fluorescence signals in the hippocampal CA1 region. However, a striking decrease in co-localization signal was observed in the GCI-Aldh1l1-Cre^ERT2^; PKM2^f/f^ group compared with the GCI-PKM2^f/f^ group (Figure 5E). In the GCI-Aldh1l1-Cre^ERT2^; PKM2^f/f^ group, LDHA and S100B co-localized fluorescence signal was significantly reduced by about 30% in the CA1 region (GCI-PKM2^f/f^, 17.3 ± 0.8; GCI-Aldh1l1-Cre^ERT2^; PKM2^f/f^, 12.0 ± 0.7) (Figure 5F). LDHB is an enzyme that is required for conversion of lactate into pyruvate in neurons. We assumed that expression of LDHB after GCI would rise to increase pyruvate and energy source production in responding to neuronal damage. However, in case of astrocyte PKM2 gene deletion, the expression of LDHB is also reduced because the amount of lactate received through the ANLS decreases. To confirm whether LDHB expression is regulated by the PKM2 gene, we used LDHB (red) and NeuN (neuron marker, green) staining after GCI. No difference in LDHB expression is observed in the sham groups. However, in the GCI groups, there was an increase in LDHB fluorescence signal in the hippocampal CA1 region. GCI-Aldh1l1-Cre^ERT2^; PKM2^f/f^ groups showed significantly reduced LDHB intensity in neurons compared with the GCI-PKM2^f/f^ groups (Figure 5G). LDHB was reduced in the GCI-Aldh1l1-Cre^ERT2^; PKM2^f/f^ groups compared with the GCI-PKM2^f/f^ groups by about 33% in the CA1 region (GCI-PKM2^f/f^, 15.2 ± 0.7; GCI-Aldh1l1-Cre^ERT2^; PKM2^f/f^, 10.2 ± 0.5) (Figure 5H).

### 3.6. Exacerbation of Neuronal Damage by PKM2 Gene Deletion Is Restored by Lactate Supplementation after GCI

**Figure 6:**
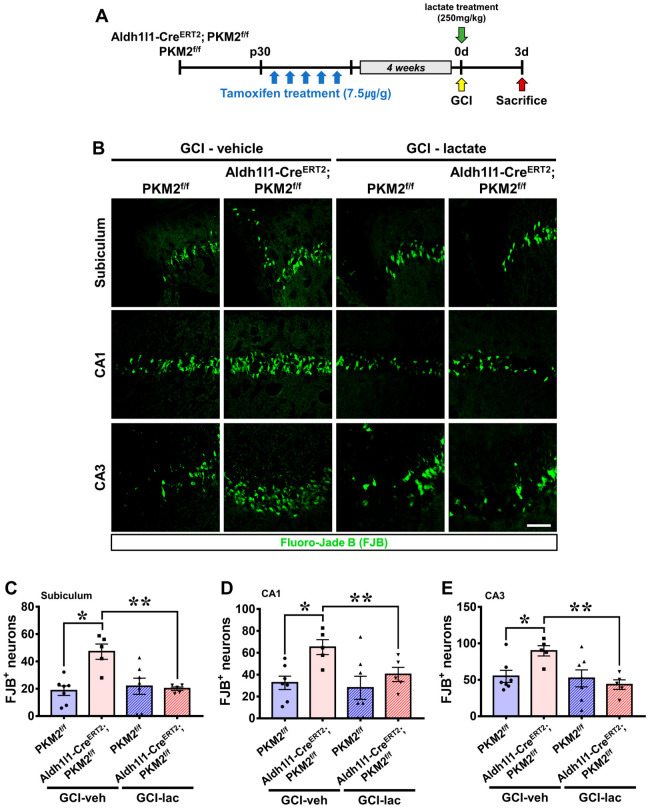
(**A**) Schematic representation showing injection of lactate immediately after GCI, and mice are sacrificed 3 days later. (**B**) Degenerated neurons in the subiculum, CA1, and CA3 regions. The GCI-lactate Aldh1l1-Cre^ERT2^; PKM2^f/f^ group showed a reduction in degenerated neurons (FJB, green) 3 days after ischemia compared with the GCI-vehicle Aldh1l1-Cre^ERT2^; PKM2^f/f^ group. Scale bar = 100 μm. (**C**–**E**) Graph indicates quantification of GCI-vehicle group compared with the GCI-lactate group (●GCI-vehicle PKM2^f/f^ group, *n* = 7; ■GCI-vehicle Aldh1l1-Cre^ERT2^; PKM2^f/f^ group, *n* = 5; ▲GCI-lactate PKM2^f/f^ group, *n* = 7; ▼GCI-lactate Aldh1l1-Cre^ERT2^; PKM2^f/f^ group, *n* = 6). *Significantly different from the GCI-vehicle PKM2^f/f^ group, GCI-vehicle Aldh1l1-Cre^ERT2^; PKM2^f/f^ group and the GCI-vehicle Aldh1l1-Cre^ERT2^; PKM2^f/f^ group, GCI-lactate Aldh1l1-Cre^ERT2^; PKM2^f/f^ group, * *p* < 0.05, ** *p* < 0.05. Data are shown as mean ± SEM (Bonferroni post hoc test after Kruskal–Wallis test, subiculum: chi square = 9.693, df = 3, *p* = 0.021; CA1: chi square = 8.572, df = 3, *p* = 0.036; CA3: chi square = 8.074, df = 3, *p* = 0.045).

Our results indicate that PKM2 plays an important role in supplying energy to neurons from astrocytes via the ANLS in the case of ischemic stroke. Therefore, we hypothesized that administration of lactate after GCI would have neuroprotective effects. We analyzed the influence of lactate administration on neuronal death after GCI by Fluoro-Jade B (FJB) staining. Lactate was injected immediately after GCI, and the responses in the brain were assessed 3 days later (Figure 6A). The GCI-vehicle groups widely showed degenerating neurons in the hippocampal regions. Consistent with our previous data, dramatically increased neuronal death was observed in the GCI-Aldh1l1-Cre^ERT2^; PKM2^f/f^ group compared with the GCI-PKM2^f/f^ group. However, fewer degenerating neurons were observed the GCI-lactate groups compared with the GCI-Aldh1l1-Cre^ERT2^; PKM2^f/f^ group. Importantly, lactate administration significantly reduced neuronal death in the GCI-Aldh1l1-Cre^ERT2^; PKM2^f/f^ group (Figure 6B). The GCI-vehicle Aldh1l1-Cre^ERT2^; PKM2^f/f^ group displayed counts of FJB+ neurons of about 60% in the subiculum (GCI-Aldh1l1-Cre^ERT2^; PKM2^f/f^, 47.1 ± 5.5; GCI-PKM2^f/f^, 18.5 ± 3.5), 50% in the CA1 (GCI-Aldh1l1-Cre^ERT2^; PKM2^f/f^, 65.2 ± 6.8; GCI-PKM2^f/f^, 32.5 ± 6.0), and 39% in the CA3 (GCI-Aldh1l1-Cre^ERT2^; PKM2^f/f^, 89.8 ± 7.0; GCI-PKM2^f/f^, 54.8 ± 8.1) regions compared with the GCI-vehicle PKM2^f/f^ groups. Then, the GCI-Aldh1l1-Cre^ERT2^; PKM2^f/f^ group, the lactate administered group showed a reduction in FJB+ neurons of about 57% in the subiculum (GCI-vehicle Aldh1l1-Cre^ERT2^; PKM2^f/f^, 47.1 ± 12.4, GCI-lactate Aldh1l1-Cre^ERT2^; PKM2^f/f^, 20.0 ± 2.9), 38% in the CA1 (GCI-vehicle Aldh1l1-Cre^ERT2^; PKM2^f/f^, 65.2 ± 15.2, GCI-lactate Aldh1l1-Cre^ERT2^; PKM2^f/f^, 40.1 ± 14.5), and 51% in the CA3 (GCI-vehicle Aldh1l1-Cre^ERT2^; PKM2^f/f^, 89.8 ± 15.7, GCI-lactate Aldh1l1-Cre^ERT2^; PKM2^f/f^, 43.4 ± 14.6) regions (Figure 6C–E).

### 3.7. PKM2 Gene Deletion Allows Recovery of Live Neurons and Neurological and Cognitive Function by Lactate Supplementation after GCI

**Figure 7:**
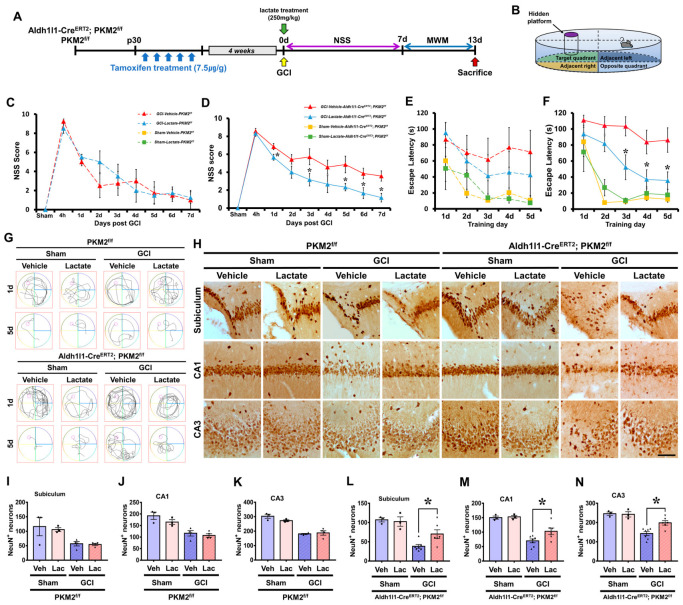
(**A**) The timeline of the experimental design with the addition of neurological severity score (NSS) and Morris water maze (MWM) test. (**B**) The scheme of the Morris water maze. (**C**,**D**) The NSS test was performed for 7 consecutive days after GCI induction in the vehicle and lactate administration groups. (**E**,**F**) MWM performance. Escape latency of acquiring trial for 5 consecutive days starting after the NSS test (*n* = 3 for each sham group, *n* = 4–7 for each global cerebral ischemia group). * Significantly different from the GCI-Vehicle-Aldh1l1-Cre^ERT2^; PKM2^f/f^, GCI-Lactate-Aldh1l1-Cre^ERT2^; PKM2^f/f^, *p* < 0.05. Data are shown as mean ± SEM (repeated measures test followed by ANOVA, the NSS test: PKM2^f/f^ group, time: F = 44.977, *p* < 0.001; group: F = 0.064, *p* = 0.809; time * group: F = 2.007, *p* = 0.077, Aldh1l1-Cre^ERT2^; PKM2^f/f^ group, time: F = 31.264, *p* < 0.001; group: F = 8.874, *p* = 0.013; time * group: F = 1.277, *p* = 0.273, MWM test: PKM2^f/f^ group, time: F = 3.877, *p* = 0.014; group: F = 0.293, *p* = 0.608; time * group: F = 0.996, *p* = 0.429, Aldh1l1-Cre^ERT2^; PKM2^f/f^ group, time: F = 16.631, *p* < 0.001; group: F = 12.328, *p* < 0.001; time * group: F = 1.984, *p* = 0.042). (**G**) The average swimming trajectory of the 8 groups during the probe trial on days 1 and 5. (**H**) Immunohistochemistry image indicating NeuN+ neurons in the subiculum, CA1, and CA3 regions. Scale bar = 100 μm. (**I**–**N**) Graph showing analysis of NeuN+ neurons in each group in the hippocampal subiculum, CA1, and CA3 regions. The GCI-lactate Aldh1l1-Cre^ERT2^; PKM2^f/f^ group shows an increase in live neurons compared with the GCI-vehicle Aldh1l1-Cre^ERT2^; PKM2^f/f^ group after GCI. (*n* = 3 for each sham group, *n* = 4–7 for each global cerebral ischemia group). (●Sham-vehicle group; ■Sham-lactate group; ▲GCI-vehicle group; ▼GCI-lactate group).* Significantly different from the GCI-Vehicle-Aldh1l1-Cre^ERT2^; PKM2^f/f^, GCI-Lactate-Aldh1l1-Cre^ERT2^; PKM2^f/f^, *p* < 0.05. Data are shown as mean ± SEM (Bonferroni post hoc test after Kruskal–Wallis test, PKM2^f/f^ group, subiculum: chi square = 5.943, df = 3, *p* = 0.114; CA1: chi square = 9.952, df = 3, *p* = 0.019; CA3: chi square = 10.524, df = 3, *p* = 0.015, Aldh1l1-Cre^ERT2^; PKM2^f/f^ group, subiculum: chi square = 11.637, df = 3, *p* = 0.009; CA1: chi square = 12.800, df = 3, *p* = 0.005; CA3: chi square = 13.730, df = 3, *p* = 0.003).

To measure neurologic and cognitive abilities, we performed a neurological severity score (NSS) and Morris water maze (MWM) test after GCI. The Aldh1l1-Cre^ERT2^; PKM2^f/f^ group, PKM2^f/f^ sham or GCI group, and each lactate administration group were tested using an MWM test for 5 consecutive days after the NSS test for 7 consecutive days. After the behavioral tests were completed, we stained NeuN to confirm that the administration of lactate increased neuron survival (Figure 7A). The MWM test consist of four trial zones, namely the target quadrant, adjacent left, adjacent right, and opposite quadrant. The hidden platform is set on the target quadrant zone. The mice performed the test while looking at the water tank wall in each zone (Figure 7B). We found that the GCI-vehicle-PKM2^f/f^ and GCI-lactate-PKM2^f/f^ groups showed no difference in the NSS score after GCI. However, significant improvement in the NSS test score was observed for GCI-induced neurological function in the GCI-Aldh1l1-Cre^ERT2^; PKM2^f/f^ lactate administration group compared with the GCI-Aldh1l1-Cre^ERT2^; PKM2^f/f^ group (Figure 7C,D). In the PKM2^f/f^ groups, mice of the sham group reached the hidden platform faster than the GCI group. However, there were no differences between the GCI-PKM2^f/f^ groups. In addition, the MWM test indicated that there was no difference between groups on days 1 and 2. However, a dramatic difference was observed in that mice of the GCI-Aldh1l1-Cre^ERT2^; PKM2^f/f^ lactate administration group reached the hidden platform faster than those of the GCI-Aldh1l1-Cre^ERT2^; PKM2^f/f^ group on days 3, 4, and 5. The average tracking record of each group on day 5 indicated that the GCI-Aldh1l1-Cre^ERT2^; PKM2^f/f^ lactate administration group reached the hidden platform significantly faster and without wandering than the GCI- Aldh1l1-Cre^ERT2^; PKM2^f/f^ group (Figure 7E–G). In addition, we addressed whether GCI-induced immediate lactate administration increases the neuroprotective effects of neurodegeneration after 13 days (Figure 7H). To determine whether the GCI-Aldh1l1-Cre^ERT2^; PKM2^f/f^ group lactate administration increased live neurons after GCI, we performed staining of numerous neuronal nuclei (NeuN)+ cells in the hippocampal regions. We showed that in the PKM2^f/f^ groups, there was no difference in each of the sham and GCI groups. In addition, there is no difference in the number of NeuN+ cells in the sham-Aldh1l1-Cre^ERT2^; PKM2^f/f^ groups, although in the GCI-Aldh1l1-Cre^ERT2^; PKM2^f/f^ groups, there is a more dramatic increase in the lactate administration group than the vehicle group. The GCI-lactate Aldh1l1-Cre^ERT2^; PKM2^f/f^ group show an increase in NeuN+ neurons of about 48% in the subiculum (GCI-lactate Aldh1l1-Cre^ERT2^; PKM2^f/f^, 38.1 ± 4.7, PKM2^f/f^, 70.1 ± 11.2), 32% in the CA1 (GCI-lactate Aldh1l1-Cre^ERT2^; PKM2^f/f^, 69.7 ± 6.7, PKM2^f/f^, 102.9 ± 11.5), and 28% in the CA3 (GCI-lactate Aldh1l1-Cre^ERT2^; PKM2^f/f^, 142.8 ± 10.6, PKM2^f/f^, 198.3 ± 11.6) regions (Figure 7I–N).

## 4. Discussion

The present study demonstrates that pyruvate kinase M2 (PKM2) gene deletion exacerbates neuronal death by reducing lactate supplementation in neurons via the astrocyte–neuron lactate shuttle (ANLS). Moreover, the administration of lactate for supplying an energy source has a neuroprotective effect, and the mice with lactate administration recover cognitive dysfunction following global cerebral ischemia (GCI). To verify whether PKM2 gene deletion increases neuronal damage after GCI and whether lactate administration repairs GCI-induced neuronal damage, we investigated neuronal death, oxidative damage, microtubule disruption, MCT4, LDHA in astrocytes, LDHB in neurons, and the delay in neuronal death and behavioral dysfunction in mice. The results show that lactate transportation from astrocytes, via the ANLS, to nearby neurons may have therapeutic potential for preventing brain damage by supplying an energy source to compensate for depleted ATP synthesis and abnormality in energy metabolism following GCI.

**Figure 8:**
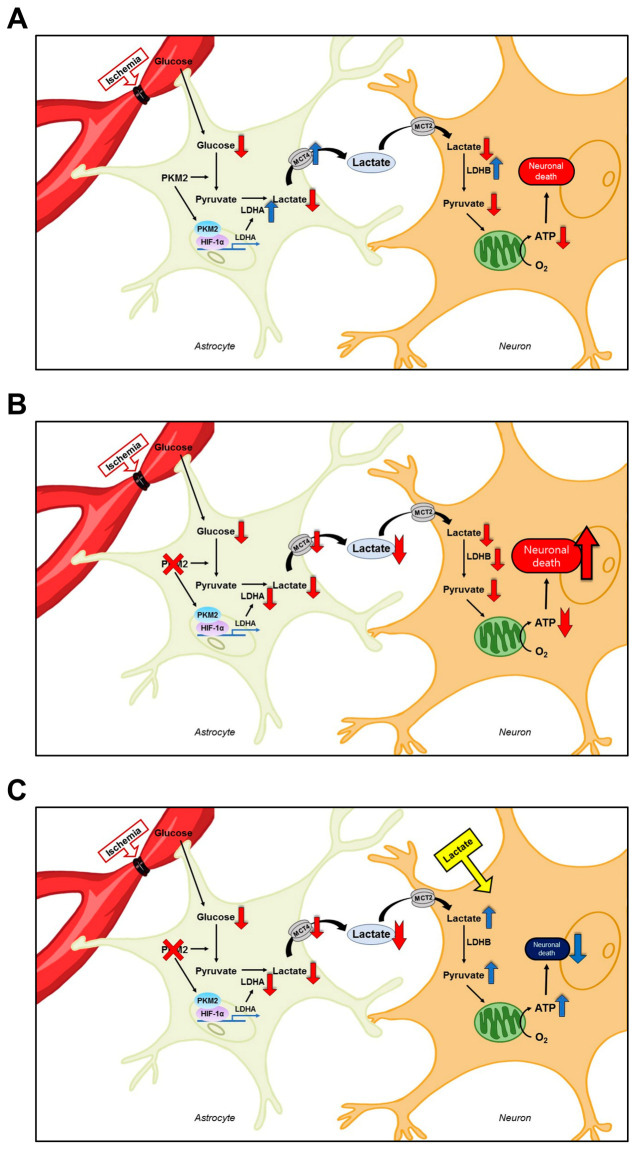
Schematic illustration showing the interaction between astrocytes and neurons via the astrocyte–neuron lactate shuttle (ANLS) after global cerebral ischemia (GCI) and lactate administration. (**A**) GCI insult reduces glucose in astrocytes and reduces lactate supply through the ANLS. As a result, neuronal death occurs, because the amount of lactate supplied decreases and the production of energy source decreases. Here, GCI-induced PKM2 with HIF-1α binding increases production of lactate dehydrogenase A (LDHA). Therefore, the expression of monocarboxylate transporters 4 (MCT4) and lactate dehydrogenase A, B (LDHB) for the production and supply of lactate to prevent neurodegeneration is also increased. (**B**) Depletion of PKM2 by astrocytic PKM2 gene deletion contributes to decreasing LDHA production. Therefore, the amount of lactate produced by the astrocyte itself decreases, which in turn reduces the amount of lactate delivered to neurons through the ANLS. As a result, a decrease in the amount of lactate received from astrocytes via the ANLS results in a relative decrease in energy source and an increase in neuronal death. (**C**) Here, instead of dysfunction the ANLS, lactate is supplemented to increase the energy source generation of neurons, which was reduced by PKM2 gene deletion. As a result, neuronal damage is reduced via increased pyruvate and energy source production in neurons by lactate administration after GCI.

Typically, astrocytes have greater metabolic plasticity and a higher capacity for glucose usage than neurons. These properties are significant for neuroprotection and homeostasis [38,39,40,41]. The high glycolysis ratio of astrocytes contributes to high lactate production and release into the extracellular space [42,43]. Lactate released by astrocytes is used by nearby neurons as energy for ATP production [44]. In the case of ischemic conditions, lactate prevents insufficient ischemia-induced glycolysis. Lactate from the ANLS involving lactate metabolism-related factors, lactate dehydrogenase A (LDHA), LDHB, and monocarboxylate transporter 4 (MCT4) increases the energy supplied to neurons from astrocytes in protecting against ischemia-induced neuronal death [1]. We hypothesized that the deletion of a specific PKM2 gene in astrocytes reduces pyruvate production. Therefore, we investigated whether astrocyte-specific PKM2 gene deletion increases neuronal death due to decreased lactate transfer via ANLS, consequently reducing production of the energy source in neurons. In addition, to overcome the lack of energy source generation by depletion of lactate in neurons after GCI, we evaluated whether the administration of lactate can prevent neuronal death (Figure 8). To confirm whether PKM2 was deleted specifically in astrocytes, we continuously injected tamoxifen in Aldh1l1-Cre^ERT2^; PKM2^f/f^ and PKM2^f/f^ mice for five consecutive days. Through PKM2 and S100B immunostaining in the hippocampal region, we found that PKM2 gene deletion reduced the intensity of PKM2-positive fluorescence signals in astrocytes. In order to confirm the difference in neuronal damage post-GCI and following PKM2 gene deletion, Fluoro-Jade B (FJB) staining was conducted to identify the hippocampal region. We found that deletion of the PKM2 gene in astrocytes significantly increased the staining of FJB+ neurons compared with PKM2 wild-type after GCI. Thus, in the present study, it is demonstrated that PKM2 gene deletion in astrocytes exacerbates neuronal death after GCI.

In our previous studies, we demonstrated that ischemia, hypoglycemia, and traumatic brain injury induce oxidative damage and disruption of microtubules in the hippocampus [3,31,32,45]. In neurodegenerative diseases, 4-hydroxynoneal, a highly reactive α,β-unsaturated aldehyde, is produced by lipid peroxidation in biological membranes [46,47,48,49]. After ischemia, ROS is increased in degenerating neurons due to NADPH oxidase activation. In the present study, we indirectly detected neuronal ROS by staining of 4-HNE as a biomarker. Astrocytic PKM2-deleted mice are unable to deliver energy sources to neurons after ischemia, resulting in increased mitochondrial damage and increased ROS generation, which aggregate neuron degeneration [50]. Microtubules play essential roles in the growth, plasticity, and differentiation of neurons. However, they appear to be the most vulnerable cytoskeletal proteins following brain ischemia [51]. To test whether oxidative damage and microtubule disruption are increased following PKM2 gene deletion and after GCI, 4-HNE and MAP-2 in the hippocampal regions were stained. The 4-HNE fluorescence signal was significantly increased in the hippocampal area, which indicates that oxidative damage-induced lipid peroxidation is increased. In addition, the MAP-2 fluorescence signal was significantly reduced in the stratum radiatum (SR) area of the hippocampal region, which suggests increased microtubule disruption.

The ANLS pathway provides neurons with lactate from astrocytes as an energy source. Previous studies have demonstrated that lactate from astrocytes is preferentially used over glucose in neurons after cerebral injury [44,52]. To verify the ANLS-related factors, we assessed LDHA and MCT4 in astrocytes and LDHB in neurons [53]. First, to confirm the expression of LDHA in astrocytes and LDHB in neurons, we performed immunostaining with LDHA, LDHB, S100B (astrocyte marker), and NeuN (neuron marker), including characterization of merged images. Then, to confirm expression of MCT4, a transporter that delivers lactate from astrocytes to extracellular space, we performed MCT4 and S100B (astrocyte) immunostaining. Global cerebral ischemia (GCI) was shown to increase LDHA, LDHB, and MCT4 expression in wild-type mice. However, significantly reduced LDHA, LDHB, and MCT4 immunostaining was observed for PKM2 gene deletion compared with the wild-type groups. Under the GCI conditions, we assume that overexpression of LDHA by binding of PKM2 with HIF-1α consequently increases MCT4 expression due to the lack of lactate production. Previous studies have shown that pyruvate can be used as a substrate for mitochondrial metabolism, which is achieved by the inverse activity of LDH, which oxidizes lactate to produce pyruvate [54,55]. However, astrocytic PKM2 gene deletion leads to greater depletion of lactate conversion from pyruvate due to reduced PKM2 with HIF-1α binding. Thus, PKM2 gene deletion-induced decreases in the expression of LDHA, LDHB, and MCT4 reveals that neuronal death is exacerbated due to the blockade of lactate transfer from astrocytes to neurons. In addition, MCT2 was more significantly reduced in PKM2 gene deletion mice after GCI. Thus, we believe that mice with PKM2 deletion have a reduced supply of lactate from astrocytes via the ANLS, which is used as an energy source for neurons. Therefore, PKM2 gene deletion contributes to the exacerbation of neuronal death due to the lack of an energy source resulting from a reduced supply of lactate through the ANLS after GCI.

Furthermore, we confirmed that exacerbation of neuronal damage by PKM2 gene deletion in the case of GCI can be restored by lactate supplementation. Previous studies have suggested that l-lactate plays a sufficient role as an energy source for neurons. Moreover, previous studies have confirmed that lactate administration increases ATP production and that lactate provides a substrate for maintaining ATP homeostasis under hypoxia conditions [54,56,57]. In addition, lactate has been demonstrated to have a neuroprotective effect on brain diseases involving ischemia and hypoglycemia [58,59,60,61]. To test whether neuronal damage is decreased by lactate supplementation, FJB was stained in the hippocampal regions after GCI. We found that lactate administration reduces neuronal damage in PKM2 gene-deleted mice. Based on these results, we suggest that lactate administration to PKM2 gene deletion mice compensates for decreased lactate supply through the ANLS, thereby reducing neuronal damage.

Finally, to show whether lactate administration had beneficial effects on neurological behavioral after GCI, we conducted tests for both the Morris water maze (MWM) and the neurological severity score (NSS). The NSS test is a scale for evaluating neurological deficit that is commonly used for brain injuries after stroke and hypoglycemia. The MWM test is one of the most common spatial learning and memory tests for animals [32,62,63,64]. In the present study, we found that the NSS increased and cognitive function decreased in PKM2 gene-deleted mice. However, lactate administration to PKM2 gene deletion mice significantly recovered the neurological severity and cognitive dysfunction even after GCI. In addition, to confirm whether live neurons were increased by lactate supplementation on day 13, we performed NeuN staining via immunohistochemistry in hippocampal subiculum, CA1, and CA3 regions after GCI. As a result, we found no differences between living neurons in the sham-PKM2^f/f^ group, GCI-PKM2^f/f^ group, and sham-Aldh1l1-Cre^ERT2^; PKM2^f/f^ group. However, live neurons were significantly increased in the GCI-lactate-Aldh1l1-Cre^ERT2^; PKM2^f/f^ group compared with the GCI-vehicle-Aldh1l1-Cre^ERT2^; PKM2^f/f^ group. This shows that the administration of lactate to the GCI-induced PKM2 gene-deleted mice supplements the reduced energy source in neurons, thereby mitigating neuronal death. Therefore, we confirmed that lactate supply via the ANLS in astrocytes, even in cases of PKM2 gene deletion, plays a beneficial role in preventing neuronal damage, oxidative stress, microtubule disruption, and neurological and cognitive dysfunction through maintaining the energy supply in neurons. We also demonstrated that lactate administration is a potential beneficial tool for preventing neuronal damage and cognitive impairment after GCI.

## Data Availability

All experimental datasets throughout the current study are available on reasonable request to corresponding author.

## Supplementary Materials

The following supporting information can be downloaded at: https://www.mdpi.com/article/10.3390/antiox12020491/s1, Figure S1: PKM2 gene deletion reduces the expression of MCT2 in the hippocampus after GCI; Table S1: Criteria of the neurological severity (NSS) in mice.
