# Supplementary material for: Effects of Pyruvate Kinase M2 (PKM2) Gene Deletion on Astrocyte-Specific Glycolysis and Global Cerebral Ischemia-Induced Neuronal Death
Source: Antioxidants (Basel). 2023 Feb 15;12(2):491. doi: 10.3390/antiox12020491 (PMC9952809; doi:10.3390/antiox12020491)
Supplement: Supplementary file 1 [file antioxidants-12-00491-s001.zip › antioxidants-2148176-supplementary.pdf]

antioxidants

Supplementary Materials

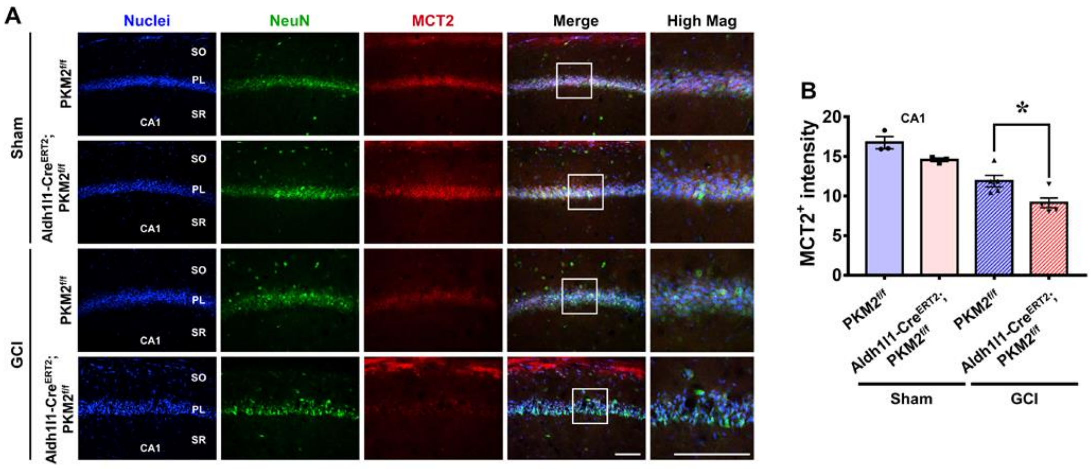

Figure S1. (**A**) the fluorescent image shows merge of monocarboxylate transporter 2 (MCT2, red), NeuN (living neurons, green) and DAPI (blue). (**B**) When averaged in the graph, there was no difference in the sham groups. However, MCT2 intensity significantly decreased with PKM2 gene deletion groups in GCI groups. (Sham-PKM2$^{f/f}$ group, n = 3; Sham-Aldh1l1-Cre^{ERT2}; PKM2$^{f/f}$, n = 3; GCI-PKM2$^{f/f}$ group, n = 5, GCI-Aldh1l1-Cre^{ERT2}; PKM2$^{f/f}$, n = 5). Scale bar = 100 ㎛. Data are shown as mean ± SEM (Kruskal–Wallis test followed by Bonferroni post–hoc test, MCT2: chi square = 12.765, df = 3, p = 0.005) *Significantly different from the GCI-PKM2$^{f/f}$ group, Aldh1l1-Cre^{ERT2}; PKM2$^{f/f}$ group, $p < 0.05$.

Table S1. Criteria of the Neurological Severity Score (NSS) in mice.

Task	Measurement	Point
1. Motor dysfunction		
Present of mono- or hemiparesis	Absent	0
	Present	1
2. Body coordination	(Unstable or drop: 1p, Criteria: 0p)	
Walk on a 3cm wide beam	Score	0-1
Walk on a 2cm wide beam	Score	0-1
Walk on a 1cm wide beam	Score	0-1
3. Body balance	(Unstable or drop: 1p, Criteria: 0p)	
Balance on a 1cm wide beam	Score	0-1
Balance on a 0.5cm diameter round stick	Score	0-1
4. Escape from 30cm diameter circle		
	Escape	0
	> 2min	1
5. General behavioral deficit		
Seeking behavior	Present	0
	Absent	1
Walk straight line	Present	0
	Absent	1
6. Startle reflex	Present	0
	Absent	1
**Total behavior score**		**10**
